# Multiplex Chromosomal Exome Sequencing Accelerates Identification of ENU-Induced Mutations in the Mouse

**DOI:** 10.1534/g3.111.001669

**Published:** 2012-01-01

**Authors:** Miao Sun, Kajari Mondal, Viren Patel, Vanessa L. Horner, Alyssa B. Long, David J. Cutler, Tamara Caspary, Michael E. Zwick

**Affiliations:** Department of Human Genetics, Emory University School of Medicine, Atlanta, Georgia 30322

**Keywords:** *N*-ethyl-*N*-nitrosourea (ENU) mutagenesis, next generation sequencing, DNA sequencing, genomics, targeted enrichment

## Abstract

Forward genetic screens in *Mus musculus* have proved powerfully informative by revealing unsuspected mechanisms governing basic biological processes. This approach uses potent chemical mutagens, such as *N*-ethyl-*N*-nitrosourea (ENU), to randomly induce mutations in mice, which are then bred and phenotypically screened to identify lines that disrupt a specific biological process of interest. Although identifying a mutation using the rich resources of mouse genetics is straightforward, it is unfortunately neither fast nor cheap. Here we show that detecting newly induced causal variants in a forward genetic screen can be accelerated dramatically using a methodology that combines multiplex chromosome-specific exome capture, next-generation sequencing, rapid mapping, sequence annotation, and variation filtering. The key innovation of our method is multiplex capture and sequence that allows the simultaneous survey of both mutant, parental, and background strains in a single experiment. By comparing variants identified in mutant offspring with those found in dbSNP, the unmutagenized background strains, and parental lines, induced causative mutations can be distinguished immediately from preexisting variation or experimental artifact. Here we demonstrate this approach to find the causative mutations induced in four novel ENU lines identified from a recent ENU screen. In all four cases, after applying our method, we found six or fewer putative mutations (and sometimes only a single one). Determining the causative variant was then easily achieved through standard segregation approaches. We have developed this process into a community resource that will speed up individual labs’ ability to identify the genetic lesion in mutant mouse lines; all of our reagents and software tools are open source and available to the broader scientific community.

Forward genetic screens in the mouse have uncovered many new features of mammalian biology. The logic of these studies is straightforward. First, a mutagen is used to randomly induce mutations in mice. Then each line is phenotypically screened to identify lines that disrupt a specific biological process of interest. Finally, a putative causative mutation within a gene is found and then directly shown to be responsible for the observed phenotype. The fruit of such studies is a collection of newly identified genes whose subsequent characterization has given us remarkable insights into mammalian biology (Caspary and Anderson 2006; Cook *et al.* 2006; Acevedo-Arozena *et al.* 2008; Beutler and Moresco 2008; Stottmann *et al.* 2011).

Although mapping and identifying a mutation using the rich resources of mouse genetics is straightforward, detecting mutations is neither fast nor cheap, thanks to two laborious bottlenecks. The first involves the fine chromosomal mapping required to localize a mutation to a small genomic region, and the second entails the Sanger-based sequencing of genes within that defined region to find putative mutations. Together, these bottlenecks discourage many from using forward genetics. Second-generation sequencing platforms hold out great promise for overcoming these bottlenecks, however, as they enable individual investigators to harness enormous raw sequencing power at a dramatically lower cost per sequenced base than traditional Sanger sequencing (Shendure *et al.* 2004; Shendure and Ji 2008). In the vast majority of mutations found to date, the mutagen induces the critical basepair change in a coding region or splice site, so sequencing can focus on these regions (exome sequencing). Several groups have successfully used deep sequencing of single-mutant individuals to identify causal mutations. The first of these groups sequenced a BAC they generated from mutant DNA; others have performed whole-exome sequencing, which enabled them to map the mutation while simultaneously detecting variants (Zhang *et al.* 2009; Fairfield *et al.* 2011; Hilton *et al.* 2011).

Despite the promise of second-generation sequencing to overcome these bottlenecks, it has yet to be broadly adopted by the mouse forward genetics community. One reason may be that the most popular mutagen used in mouse forward genetics, ENU, induces point mutations, so when sequencing is used in these lines, two challenges arise: (1) distinguishing the ENU-induced variants from strain polymorphisms and (2) identifying real variants (either ENU-induced or strain-specific) from technical sequencing errors. Here we investigate how to best surmount these challenges.

From our experience performing recessive forward genetic screens, we have made several observations with a practical impact on our strategy for overcoming these sequencing challenges. In the course of our screens (schematic in Figure 1A), we look for phenotypes of interest in the generation 3 (G3) litters (Horner and Caspary 2011). We define a line once we observe the phenotype in a quarter of the embryos across three litters *and* identify at least one G2 male. The latter requires us to set up blind crosses as we cannot genotype for the unknown mutation, meaning only one out of every four crosses results in our seeing the phenotype. Practically, this means that by the time we establish a line, we have at least four litters, usually more. Because each litter averages eight embryos, of which around two individuals are affected, we can easily determine chromosomal linkage by genome scanning four affected individuals. This allows us to genotype potential carriers in the mouse room, which means immediate and dramatic reductions in mouse costs.

**Figure 1:**
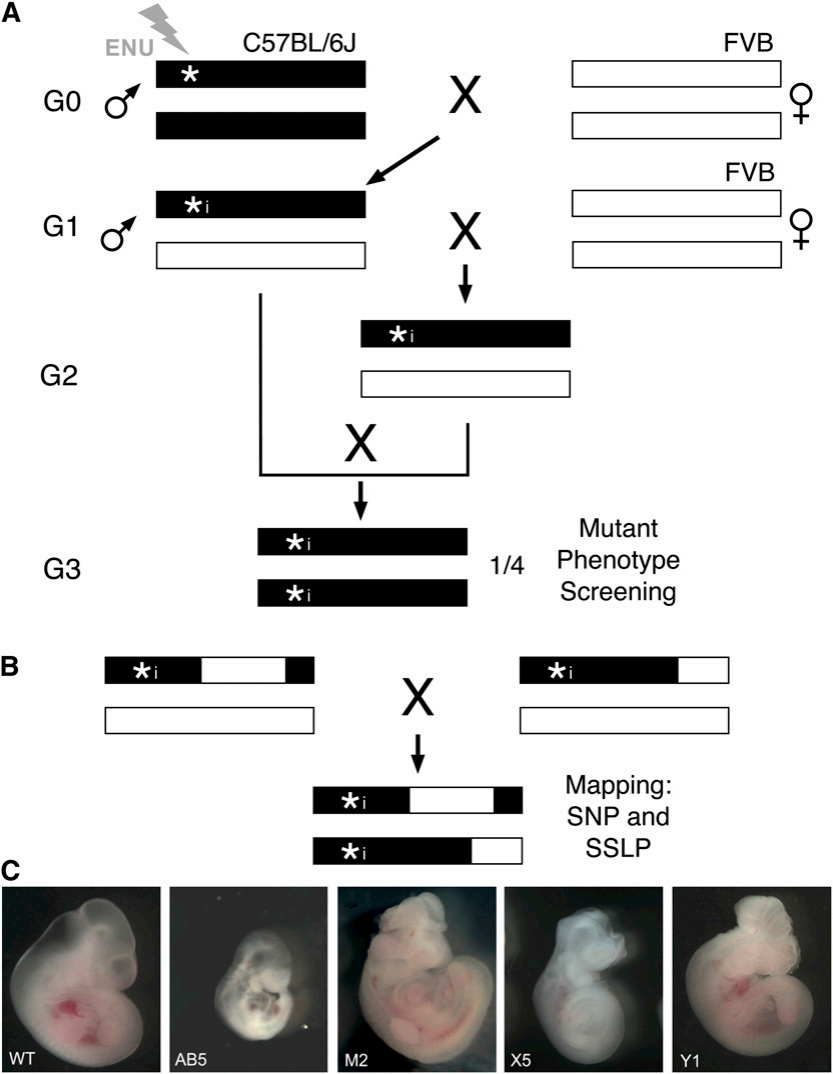
ENU mutagenesis screen and mapping. (A) ENU-mutagenized C57BL/6J male mice (generation 0, G0) were crossed to FVB/NJ female mice to generate G1 males, which were again crossed to FVB/NJ female mice. G2 females were backcrossed to their respective G1 fathers to produce G3 embryos, which were screened for neural phenotypes. Asterisks (*) indicate ENU-induced mutations, “i” next to asterisk (*i) shows the inherited ENU-induced mutation, and black and white lines depict C57BL/6J and FVB/NJ. (B) The causative mutation segregates with the C57BL/6J DNA, whose interval can be defined by linked polymorphic markers. (C) E10.5 wild-type (WT) and mutant embryos (AB5, M2, X5, and Y1) identified in ENU screen.

As we routinely establish chromosomal linkage of our lines, second-generation sequencing offers us at least two strategies: (1) to sequence genome-wide and ignore the data from unlinked chromosomes or (2) to sequence in the linked region more thoroughly. We chose the latter, as we could take advantage of the recent development and validation of methods of isolating target DNA from complex eukaryotic genomes that can be easily sequenced on second-generation platforms [Bashiardes *et al.* 2005; Albert *et al.* 2007; Fredriksson *et al.* 2007; Hodges *et al.* 2007; Okou *et al.* 2007, 2009; Porreca *et al.* 2007; Bau *et al.* 2009; Gnirke *et al.* 2009; Ng *et al.* 2009, 2010; Nikolaev *et al.* 2009; Tewhey *et al.* 2009a, 2009b; Turner *et al.* 2009; Bainbridge *et al.* 2010; Patel *et al.* 2010; and review in Mamanova *et al.* (2010)]. Furthermore, for the same cost as a single sample, we pursued a multiplex approach that let us both capture and then sequence multiple samples simultaneously. Because we know the inheritance pattern of any phenotype-causing mutation in a genetic screen, we reasoned it should be possible to distinguish strain and sequencing variation by precisely defining a putative mutation as a *de novo* variant that occurred at expected ratios in homozygous and heterozygous individuals, but in neither background strain. Thus, we were able to validate a method that exploits advances in isolating target DNA and next-generation sequencing with an added comparative approach in a cost-effective manner.

We found this strategy greatly enhanced our ability to find the most likely causal variant. Here we show that the major benefit of our multiplexed approach is to eliminate from consideration variants resulting from strain polymorphisms and technical errors. We took four novel ENU lines we had identified in a recent ENU screen, and for each line, we analyzed six DNA samples, including one from a previously identified ENU-induced line (positive control), an affected mutant embryo, each of the affected embryo’s parents, and each of the original background strains used in the screen. In all four lines examined, this approach dramatically reduced the number of candidate mutations *vs.* what we would have found had we used the data from the affected individuals only. Thus, for each of the four lines, we now have strong candidate lesions that segregate with the relevant phenotype. Notably, we found that the sequence comparison between the affected individuals and the background strains we used dramatically reduced the number of putative mutations, arguing that simplex whole-exome approaches would also benefit from this comparison.

## Materials and Methods

### Mouse screen

We performed a recessive ENU mutagenesis screen as previously described (Garcia-Garcia *et al.* 2005; Horner and Caspary 2011). Briefly, we mutagenized C57BL/6J mice and crossed them to FVB/NJ. We examined G3 embryos for abnormal development of the nervous system at embryonic day 10.5 (E10.5). Of 121 lines screened, we found 11 lines with defects in brain and spinal cord morphology. Through a genome scan using single nucleotide polymorphism (SNP) or simple sequence length polymorphism (SSLP) markers that differ between the C57BL/6J and FVB/NJ strains, we mapped four lines to defined intervals on individual chromosomes.

### Mouse samples used in our study

Six samples were used for each of the microarray-based genomic selection (MGS) experiments. Samples used for each line included two genetic background strains (C57BL/6J and FVB/NJ), one mouse homozygous for the mutant allele, and its two parents heterozygous for this allele. For each chromosome, we included the relevant positive heterozygous control from one of three known strains: *Rab23^opb2^*, *Nckap1^khlo^*, or *Ift172^wim^* on chr1, chr2, and chr5, respectively (Eggenschwiler *et al.* 2001; Huangfu *et al.* 2003; Rakeman and Anderson 2006).

### Design of custom microarrays for microarray-based genomic selection

We used a software package named MOPeD (Microarray Oligonucleotide Probe Designer, http://moped.genetics.emory.edu/) to design all MGS microarrays for this project (Patel *et al.* 2010). This web-based software allows individual investigators to easily design custom genome capture arrays that have been optimized for maskless array synthesis by Roche NimbleGen. We used the existing mouse chr1, chr2, and chr5 MOPeD-designed MGS arrays for genomic selection and obtained the target sequences for the mouse chromosomes from the UCSC genome browser RefSeq Genes track (mm9 build). We targeted all the coding and noncoding exons within the region of interest. Non-overlapping fragments were padded with 100 bp on either side to ensure proper capturing of splice sites and fragment ends. For the chr1 chip, we included the exome between the MIT markers D1MIT251 and D1MIT132, where AB5 mapped, as well as all exons of the positive control *Rab23*. For the chr2 chip, we included the exome between the MIT markers D1MIT81 and D2MIT152 (candidate region for M2) and between D2MIT285 and D2MIT411(candidate region for X5), as well as all exons of the positive control gene *Nckap1*. For the chr5 chip, we included the exome between the MIT markers D5MIT361 and D5MIT20, where Y1 mapped, as well as all exons of the positive control gene *Ift172*.

### Performing microarray-based genomic selection

MGS was carried out as described previously (Okou *et al.* 2007, 2009; Patel *et al.* 2010) with limited protocol modifications (described below). We first fragmented 5 µg of genomic DNA (mean size 300 bp, range 200–600 bp) from each of the six different mice, then repaired the ends and ligated distinct Illumina multiplex adaptors to each sample. Thus, each mouse sample had a unique multiplex adaptor tag that allowed simultaneous hybridization to a single MGS array. The sequence tag allowed us to deconvolute the sequences and, therefore, to relate the sequence to a specific mouse sample after Illumina sequencing. To ensure equal representation of multiplex adaptor-tagged DNA fragments from each of the six mice, we performed quantitative PCR with a probe complementary to the Illumina adaptors to accurately assess the quantity of tagged fragments for each sample prior to hybridization on the MGS array. We then pooled 166.6 ng from each of the six samples (total 1 µg) and hybridized this pool to the MGS array using the standard protocol. The remaining steps, which include eluting and sequencing the product, were done via our previously described standard protocol (Okou *et al.* 2009; Patel *et al.* 2010).

### Illumina sequencing and data analysis

After MGS, the enriched DNA samples were quantitated using qPCR, and then were denatured and diluted to a concentration of 8 pM. Each enriched library (having six samples with different index tags) was sequenced in a single lane of an Illumina HiSequation (100 bp, multiplexed, paired end for chr5; 100 bp, multiplexed, single end for chr1 and chr2). After sequencing, the reads were mapped against the reference sequences for respective regions using Emory Mapper (D. J. Cutler and M. E. Zwick, personal communication), and variants sites were identified. Those variants were then annotated using SeqAnt (Shetty *et al.* 2010). Next we used custom Perl scripts to process the SeqAnt output files to first identify all unique variants (Seqant.parse.pl), and then to identify and count single nucleotide variants (SNV) that were homozygous in the mutant individual and not found in dbSNP or the background strains (Seqant.count.pl), and finally to list the genotypes inferred from sequence data for the parents and mutant offspring (Seqant.genotype.pl).

### Sanger sequencing

We verified the replacement variants we identified that were within the genomic interval linked to the phenotype through Sanger sequencing. For each variant, we amplified the region surrounding the variant from genomic DNA from two mutant embryos (primers in supporting information, Table S2).

### Pedigree genotyping

For the top six candidate replacement variants, we found they either created or destroyed a restriction enzyme polymorphism that we could use to genotype directly for the variant. The enzyme used to detect the polymorphisms and their respective genes were: *Hae*III (*Ankrd56*), *Hae*III (*Inpp5e*), *MluC*I (*Slc2a6*), *Sfc*I (*Rqcd1*), *Mob*I (*Rbm12*), and *Mob*I (*Duspd15*). We genotyped all embryos from between 32 and 41 litters in each line for the variant. In the course of the screen, we found “abnormal” embryos that resembled neither the mutant nor the wild-type phenotype (Table S2). This was likely due to both spontaneous abnormal development and the high mutation load in the screen. Consistent with these ideas was our finding of no correlation between any variant and any “abnormal” phenotype in these embryos, suggesting the abnormal embryos were not the result of the variants we identified.

## Results

### Forward genetic screen and mapping

We performed a recessive ENU mutagenesis screen to identify genes important in the development of the mouse nervous system. We induced mutations on a C57BL/6J background and crossed to an FVB/NJ background, as shown in Figure 1A. By examining G3 embryos, we identified four mutant lines, AB5, M2, X5, and Y1, which displayed abnormal neural morphology at E10.5 (Figure 1C). These phenotypes were inherited in an autosomal recessive fashion with complete penetrance. AB5 mutant embryos were posteriorly truncated and showed abnormal brain morphology and cranioedema. M2 mutant embryos exhibited midbrain and hindbrain exencephaly, a dysmorphic neural tube, and anophthalmia or microphthalmia. X5 mutant embryos showed a general developmental delay along with an open forebrain and midbrain. Y1 mutant embryos displayed whole brain exencephaly and an undulated neural tube. We genotyped 4 affected G3 embryos using the Illumina mouse low-density linkage panel for SNP mapping in order to map the location of each putative recessive mutation to an individual chromosome (Moran *et al.* 2006). We found AB5 was linked to chr1; M2 and X5 were both on chr2; and Y1 was located on chr5.

### Targeted sequencing and mutation discovery

**Figure 2:**
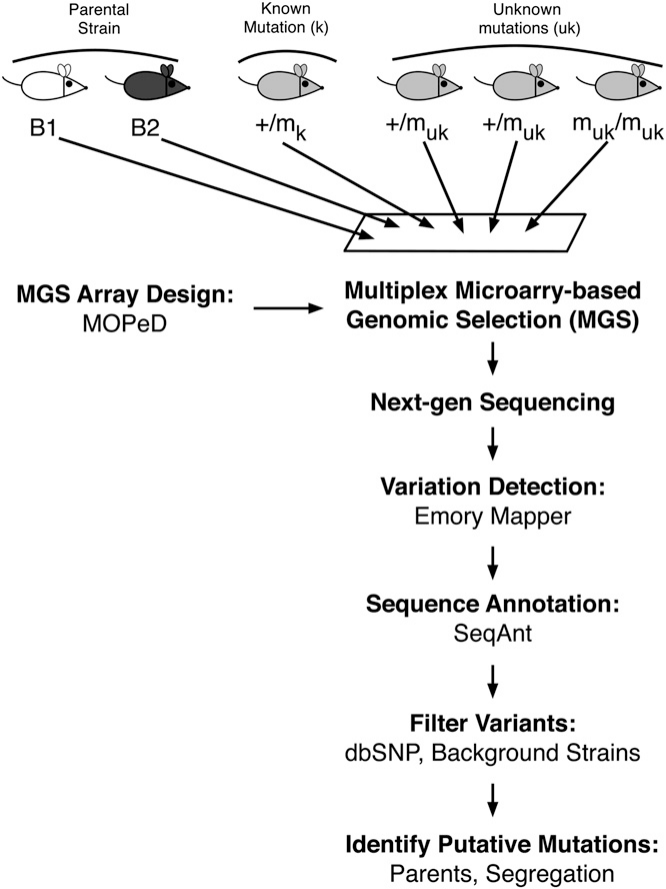
Sequencing and analysis pipeline: identifying ENU-induced mouse mutations with chromosome-specific exome sequencing. DNA samples from an affected embryo (m_uk_/m_uk_), the affected embryo’s parents (+/m_uk_), unmutagenized individuals from the two background laboratory strains (B1 and B2), and a positive control known mutation (+/m_k_) were multiplexed through our targeted sequencing pipeline to validate our approach and identify novel mutations.

Using MOPeD to tailor existing mouse chromosome exome designs, we performed targeted enrichment of all exons and adjacent intronic sequences contained within the mapped boundaries for each of the four mutant lines (Patel *et al.* 2010). Our previous analysis of single mouse samples showed we obtained far more sequence depth than needed to identify a known mutation in a single lane of Illumina sequencing. To speed the discovery of unknown ENU-induced mutations, we reasoned that, for the same basic cost, we could simultaneously sequence six distinct samples on a single microarray during the enrichment step (multiplexing). These samples included an affected embryo, its two heterozygous parents, one unmutagenized individual from each of the two background strains used in the screen, and as a positive control, a distinct, known ENU-induced line that mapped to the same chromosome. We performed multiplex MGS followed by sequencing on an Illumina platform for each of the four mutant lines (Figure 2). After mapping sequence reads, approximately 90% of targeted regions had a sufficiently high coverage (>8X) to identify variant sites for subsequent analysis. Comparable coverage was obtained for each of the multiplexed lines sequenced. The complete distribution of sequence coverage is shown in Figure S1.

A causative mutation that explains the phenotype we observed had to be found as homozygous within the mutant line and be newly induced by the action of the ENU mutagen. To determine whether such a variant was found within each of our multiplex lines, we first functionally annotated variants for each line using SeqAnt (Shetty *et al.* 2010). We then cataloged all homozygous variant sites by functional class for each of the four mutant lines (Table 1, column 3). We then filtered these variants in two ways. First, because our mutation would be ENU induced, we expected it should not be a common polymorphism contained within dbSNP. Applying this filter to all the homozygous variant calls for all four mutant lines eliminated 58% (1179 of 2043) of variant sites from further consideration (Table 1, column 4). Second, as the newly induced mutation should not be found in either of the laboratory background strains (C57BL/6J or FVB/NJ) used, we next ruled out variants found in these strains from further consideration. In total, this allowed us to eliminate an additional 40% (816 of 2043) of variants from further consideration (Table 1, column 5). The remaining 2% (48 of 2043) of variants should thus include mutations that explain the mutant phenotypes we observed (Table 1, column 6). Among the remaining 48 variant sites, only 15 were replacement variants, which constituted the class most likely to harbor the mutant alleles. Hence our two-stage screen eliminated 99.3% (2028 of 2043) of variant sites, allowing us to focus on the 0.7% (15 of 2043) of sites across the four lines that were most likely to be the causative alleles we sought.

**Table 1:** Results of filtering homozygous variants sites for each mutant line sequenced

Mutant Line	Functional Classes	Total Homozygous Variants	In dbSNP	In Background Strains, Not in dbSNP	Remaining Putative Mutations
AB5	Replacement	96	80	13	3
AB5	Silent	157	143	12	2
AB5	UTR	331	191	135	5
AB5	Intronic	106	87	17	2
AB5	Intergenic	54	50	4	0
M2	Replacement	43	8	31	4
M2	Silent	19	11	7	1
M2	UTR	73	16	55	2
M2	Intronic	46	18	20	8
M2	Intergenic	40	4	36	0
X5	Replacement	128	59	63	6
X5	Silent	192	128	63	1
X5	UTR	387	231	152	4
X5	Intronic	205	116	86	3
X5	Intergenic	89	34	55	0
Y1	Replacement	17	1	14	2
Y1	Silent	5	0	4	1
Y1	UTR	14	2	11	1
Y1	Intronic	34	0	31	3
Y1	Intergenic	7	0	7	0

### Identification of mutant alleles

Mapping of the mutant lines during the course of stock maintenance reduced the size of the region harboring the mutant alleles, allowing us to eliminate an additional nine replacement variants from further consideration (Table 2). Two of the lines (AB5 and Y1) had only a single candidate mutation, whereas another two (M2 and X5) had two candidate mutations each. We confirmed all six replacement variants by Sanger sequencing (data not shown). We genotyped each variant within each line’s existing pedigree and calculated the probability that the observed correlation of the phenotype-genotype segregation pattern was due to chance (Table 3). In all six cases, the variants segregated with the respective phenotypes.

**Table 2:** Candidate replacement variants in four mutant lines

Mutant Line	Remaining Replacement Variants	Replacement Variant(s) Within Mapped Region	Candidate Mutation(s)
AB5	3	1	Rqcd1(L159^*^)
M2	4	2	Inpp5e (D511G)
			Slc2a6(M99I)
X5	6	2	Rbm12(T556I)
			Dusp15(I157F)
Y1	2	1	Ankrd56(S299P)

**Table 3:** Segregation data for candidate mutations

Mutant Line	Candidate Mutation	Phenotype	Observed Genotype	Number of Mice	Probability
AB5	Rqcd1(L159^*^)	Wild-type	T/A or T/T	201	4.7 × 10^−58^
		Wild-type	A/A	0	
		Mutant	T/A or T/T	0	
		Mutant	A/A	57	
M2	Inpp5e(D511G)	Wild-type	C/T or T/T	254	1.3 × 10^−74^
		Wild-type	C/C	0	
		Mutant	C/T or T/T	0	
		Mutant	C/C	80	
M2	Slc2a6(M99I)	Wild-type	C/A or C/C	254	1.3 × 10^−74^
		Wild-type	A/A	0	
		Mutant	C/A or C/C	0	
		Mutant	A/A	80	
X5	Rbm12(T556I)	Wild-type	A/G or G/G	161	6.7 × 10^−49^
		Wild-type	A/A	0	
		Mutant	A/G or G/G	0	
		Mutant	A/A	55	
X5	Dusp15(I157F)	Wild-type	T/T or T/A	159	3.4 × 10^−42^
		Wild-type	A/A	2	
		Mutant	T/T or T/A	4	
		Mutant	A/A	51	
Y1	Ankrd56(S299P)	Wild-type	A/G or A/A	196	1.1 × 10^−53^
		Wild-type	G/G	0	
		Mutant	A/G or A/A	0	
		Mutant	G/G	42	

In the AB5 mutant line, we found a single T-to-A transversion in exon 4 of *Rqcd1*. This change is predicted to result in a leucine being converted to a stop codon. Among 33 litters examined, all 46 embryos with the mutant AB5 phenotype were shown to possess the A/A genotype, suggesting it is causal (Table 3). Providing further evidence for this conclusion is the observation that the *Rqcd1* transcript is found in an expressed sequence tag (EST) library generated from E8.0 whole-mouse embryo, such that the gene is expressed just prior to when we observed morphological embryonic anomalies.

For line M2, we found two potential causal variants in genes that lay 0.63 Mb apart within the candidate interval: a T-to-C transition in *Inpp5e* and a C-to-A transversion in *Slc2a6*. Both mutations are nonconservative: the transition in *Inpp5e* changed an aspartic acid to a glycine, and the transversion in *Slc2a6* altered a methionine to an isoleucine. Among 41 M2 litters examined, we found that all M2 mutants genotyped were homozygous for the C/C *Inpp5e* and the A/A *Slc2a6* variants, whereas wild-type embryos that appeared wild-type were never homozygous for the newly induced alleles (Table 3). Furthermore, there are EST clones representing both genes in mouse embryonic brain libraries. Taken together, both variants remain candidate causal mutations for the observed mutant phenotype. Notably, *Inpp5e*-null mice have been characterized and share several aspects of the M2 phenotype, the most obvious being the exencephaly we see in both the M2 and *Inpp5e*-null mice (Jacoby *et al.* 2009). This correlation suggests that the M2 phenotype may be caused in large part by the *Inpp5e* mutation we uncovered. The contribution, if any, of the *Slc2a6* variant to the M2 mutant phenotype awaits more careful phenotypic characterization of the M2 line, as well as either another *Slc2a6* allele or a recombination event that eliminates *Slc2a6* from the candidate interval.

We also found two potential variants at two loci for the X5 line. Within the X5 candidate interval, we found a G-to-A transition in *Rbm12* and a T-to-A transversion in *Dusp15*. These are predicted to change amino acid 556 of *Rbm12* from threonine to isoleucine and amino acid 157 of *Dusp15* from isoleucine to phenylalanine. We identified EST clones of *Rbm12* from E8.0 embryonic libraries, but we found no evidence for embryonic *Dusp15* expression in the EST databases. Upon genotyping 32 litters from the X5 pedigree, we saw that mutant animals were always homozygous A/A at *Rbm12* (Table 3). In contrast, we found four X5 mutant embryos that were heterozygous (T/A) for the *Dusp15* variant. Additionally, we detected two apparently wild-type embryos that were homozygous (A/A) for the *Dusp15* variant. Thus our genotype data provide greater support for the variant at *Rbm12* *vs.* the variant at the *Dusp15* (Table 3). Although our findings might be explained by incomplete penetrance of alleles at *Dusp15*, the most parsimonious explanation is that a recombination event took place, excluding *Dusp15* from the candidate interval. Taken together, we conclude that the variant in *Rbm12* is most likely to cause the X5 phenotype.

For line Y1, we identified an A-to-G transition in the single exon gene *Ankrd56*, which is predicted to change serine 299 of the protein to proline. We analyzed 33 litters from the Y1 pedigree and found all of the phenotypic Y1 mutant embryos genotyped G/G, whereas the nonphenotypic embryos were A/A or G/A (Table 3). We also found EST clones of *Ankrd56* in E8.0 libraries. In total, the strong genotype-phenotype correlation, the fact that the newly induced variant results in a nonconservative amino acid change, and the pattern of expression of the *Ankrd56* locus all argue that this variant is likely to underlie the Y1 phenotype.

In all four lines, we found that the putative causative mutations were homozygous in the affected mutant embryos and called heterozygous in their parents in the initial sequencing experiment, as expected from the recessive inheritance of the mutation. The only exception was *Dusp15*, where one parent had very poor depth of coverage and was not called a heterozygote at the relevant site.

## Discussion

Currently, the unparalleled insight into fundamental biological processes afforded by phenotype-based screens in the mouse is undermined somewhat by the challenges in identifying the affected gene. Here we accelerate the discovery of likely causative mutations in four novel ENU-induced lines using a methodology that combines multiplex chromosome-specific exome capture, next-generation sequencing, rapid mapping, sequence annotation, and variation filtering. Although additional formal genetic experiments are required to definitively demonstrate that we have identified the causative variant, the findings presented here are strong evidence that the replacement variants we found are the best candidates. Given that each line has only one or two candidate genes in need of follow up, integrating our approach more broadly into forward genetic screens in the mouse has the potential to speed up the gathering of functional information about the mammalian genome.

Whole-exome and whole-genome sequencing with next-generation platforms have been used to successfully identify mutations in the mouse (Zhang *et al.* 2009; Arnold *et al.* 2011; Fairfield *et al.* 2011; Hilton *et al.* 2011). Here we show that by using targeted sequencing of multiple samples, we can reduce the number of candidate causative mutations to one or two per line. We believe our approach offers a number of advantages compared with simplex exome sequencing of mutant individuals. First, mapping a newly induced mutation to specific chromosomes is inexpensive and routinely conducted, regardless of the type of follow-on sequencing performed. Given the ease and low cost of linkage mapping a newly arising mutation to a chromosome, as well as the reduction in mouse costs, the seemingly most natural and efficient strategy for finding newly induced mutations would focus sequencing effort solely on those chromosomes that linkage indicates must harbor a newly induced mutation. Second, multiplexing six samples on a single MGS microarray reduces the cost of capture reagents, while providing valuable data vital to pinpointing the causative mutations. Furthermore, focusing on single chromosomes reduces sequencing costs and the bioinformatics burden associated with whole-exome/whole-genome datasets. Strikingly, we found that by including the background strains in the selection and sequencing, we eliminated all but a handful of variants sites from consideration, making the identification of the putative mutation dramatically faster. The composition of those eliminated variants presumably includes both true variant sites that are unique to a given laboratory mouse strain and false-positive variant sites that arise due to systematic mismapping and/or imperfections in genotype calling of next-generation sequencing data. In the case of a simplex whole-exome/whole-genome sequencing design, where only the affected individual is sequenced, these additional variants would have to be pursued with another technology, like Sanger sequencing, thereby slowing identification of the putative mutation. In theory, the same approach of using the identical sequencing platform for the background strains could be used for simplex whole-exome analysis. However, even as sequencing costs continue to decline, the costs of capture reagents for each additional line are fixed.

Published approaches to identifying causative mutations routinely rely on screening out variants found in dbSNP (Fairfield *et al.* 2011; Hilton *et al.* 2011). We found that doing so eliminated 58% of the observed variants. Strikingly, an additional 40% of our detected variants appear to be real polymorphisms present in our laboratory’s parental strain or technical artifacts not currently seen in dbSNP. In contrast, our inclusion of the heterozygous parental lines only allowed us to screen out a handful of sequencing errors that appeared homozygous in the affected mutant embryo but were unobserved in the parents. Our demonstration of the power gained by sequencing the background strain using the same sequencing platform as the affected mouse is important, as it presumably needs to be done only once at a particular laboratory site. Thus, individual investigators could in effect have a personalized database of sequence variants for their background strains in addition to those found in public databases like dbSNP. Given such a resource, our design could be further modified to multiplex affected individuals from distinct lines, which would further reduce the cost per mutant line.

At a molecular level, the clear advantage of using sequencing is evident in the M2 line, where two replacement mutations remain candidates. Using traditional methods, one can easily imagine narrowing the genomic interval to the same degree and seeing that the characterized null phenotype of *Inpp5e* mimics that of M2. Should M2 fail to complement the *Inpp5e*-null allele, we would never realize that *Slc2a6* also carries a potentially functional variant. Similarly, should neither the *Inpp5e* nor the *Slc2a6* variants underlie the M2 phenotype in formal genetic tests, we already know the two UTR variants that would be the next best candidates (Table 3). Thus, comprehensive targeted sequencing enables the identification of all possible mutations that will give us a more thorough understanding of alleles in the future.

Finally, although we chose to use custom-designed microarrays as our selection platform because of their easy availability and lower cost, they do require some standard hardware to process the microarrays. Chromosome-specific liquid capture may be another viable strategy that could be pursued in the future. Our custom microarray designs, which were not empirically optimized, are freely available for use by the broader community of mouse geneticists. Using our data from this experiment will allow immediate improvement in the design of such arrays to further improve the performance of the targeted enrichment for future sequencing studies.

The cost savings of our method are clear, albeit difficult to calculate precisely. The biggest fixed expense in performing forward genetics is the “cage costs” associated with breeding. Therefore, the initial chromosomal linkage of newly induced mutations is cost effective, because it reduces the number of crosses one needs to set up by 75%. While sequencing costs continue to drop precipitously, our method provides a means to verify results across multiple individuals, while generating sufficient sequencing coverage for each individual sample. The result is that we identify only a few real candidate variants, which inherently reduces the costs associated with following up potential variants. Taken together, our data show that we can now more efficiently pinpoint the most likely causal variants in ENU-induced lines.

### Implications for mouse forward genetics

One valid criticism of forward genetic screens is that, in addition to identifying novel genes, one can easily invest a great deal of time discovering a new allele, or even rediscovering a previously identified allele, of an already characterized gene. For example, the mutation we identified in the M2 line likely represents a new *Inpp5e* allele. Historically, the existence of allelic series of specific loci has proved invaluable. This is especially true for structure-function studies, as well as for understanding the effects of mutations found in humans. Distributing new alleles of previously characterized genes through the regional mouse mutant resource centers could be of great value to the mouse community at large. Still, as one of the main goals of a forward genetic screen is to identify *novel* loci that govern basic biological processes, there are clear advantages to the investigator being able to choose which lines to work on knowing whether the gene has previously been characterized. Our method has the potential to change the way the forward genetics community conducts screens, as we would be able to first systematically find the molecular lesions in all lines and then integrate that information in choosing which lines to pursue. Our data argue that such an ideal is within reach.

## Supplementary Material

HTML Page - index.htslp
