# Supplementary material for: Multiplex Chromosomal Exome Sequencing Accelerates Identification of ENU-Induced Mutations in the Mouse
Source: G3 (Bethesda). 2012 Jan 1;2(1):143–50. doi: 10.1534/g3.111.001669 (PMC3276189; doi:10.1534/g3.111.001669)
Supplement: HTML Page - index.htslp [file supp_2_1_143__index.html]

HTML Page - index.htslp 

# Multiplex Chromosomal Exome Sequencing Accelerates Identification of ENU-Induced Mutations in the Mouse

## HTML Page - index.htslp

**Files in this Data Supplement:**

- Supporting Information
- Figure S1 - Complete distribution of sequence coverage (pdf, 308 KB)
- Table S1 - Primer sequences used for Sanger sequencing and verification of variant sites (pdf, 68 KB)
- Table S2 - Summary of embryos genotyped (pdf, 64 kb)
