# Supplementary figures and images for: Multiplex Chromosomal Exome Sequencing Accelerates Identification of ENU-Induced Mutations in the Mouse
Source: G3 (Bethesda). 2012 Jan 1;2(1):143–50. doi: 10.1534/g3.111.001669 (PMC3276189; doi:10.1534/g3.111.001669)

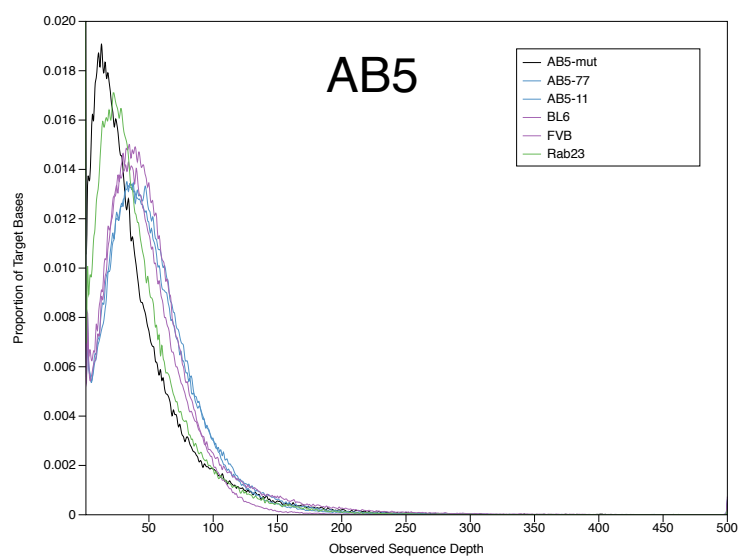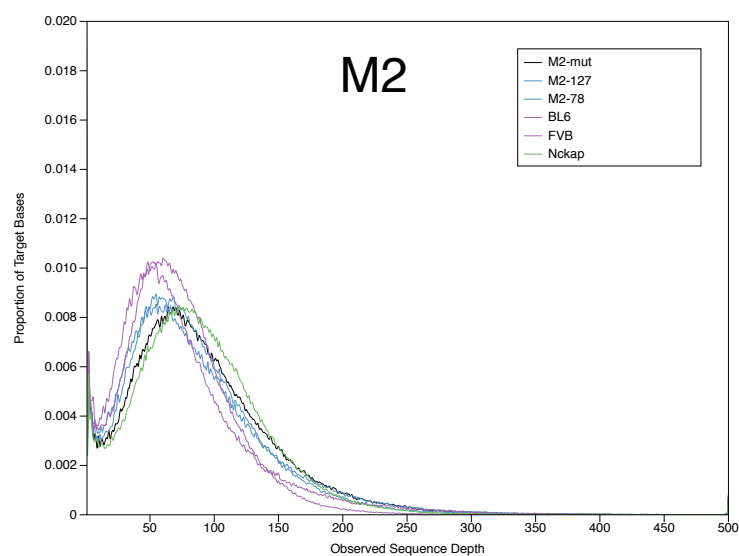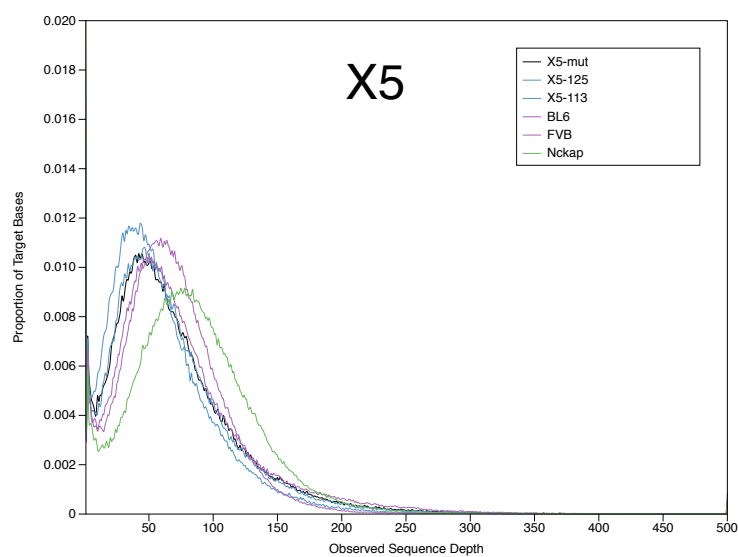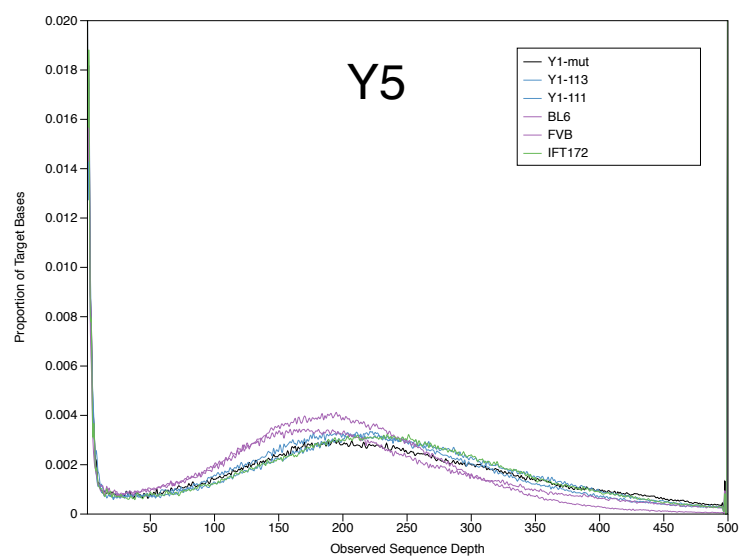

**Figure S1** Complete distribution of sequence coverage.

Supplement: HTML Page - index.htslp [file supp_2.1.143_FigureS1.pdf]
