# Supplementary material for: Multiplex Chromosomal Exome Sequencing Accelerates Identification of ENU-Induced Mutations in the Mouse
Source: G3 (Bethesda). 2012 Jan 1;2(1):143–50. doi: 10.1534/g3.111.001669 (PMC3276189; doi:10.1534/g3.111.001669)
Supplement: HTML Page - index.htslp [file supp_2.1.143_TableS1.pdf]

Table S1 Primer sequences used for Sanger sequencing and verification of variant sites

Locus	Forward Primer (5'-3')	Reverse Primer (5'-3')	Start	Stop
Ankrd56	CTGAGGACTCATCGAGAACCACGGG	CGAGCACGCGCCTGGATCTG	Chr5:93472858	Chr5:93473240
Inpp5e	CTGCAACACAGGAGAGTCAGGGAGG	ATCAGAGCAGATGAGGGGAGGCCAG	Chr2:26254869	Chr2:26255149
Slc2a6	ACCTCTGCCAACAGGGGACTCTACC	CGTCAGCATCCTCCCCAACAGAAGC	Chr2:26881457	Chr2:26881752
Slc2a6-MluCI	GCAGGGGGCCTCAGTGCAAT	CGTCAGCATCCTCCCCAACAGAAGC	Chr2:26881571	Chr2:26881752
Rbm12	CCAATGCTTGCCCTAGACCTTGCCC	GGACCCAATGGGAAAGCAACTGGTG	Chr2:155922319	Chr2:155922694
Rbm12-MobI	CCAATGCTTGCCCTAGACCTTGCCC	CATATAACAAACATTCCATTCAGCATGA	Chr2155922319	Chr2: 155922448
Rqcd1	GGATTCCCTGACACAGAAACTACAG	AAGCACGCAGTACCGTTTTAGAGAG	Chr1:74569954	Chr1:74570200
Rqcd1-SfcI	AGATGAGCAAGAAGTAATCAACTTTCTAT	AAGCACGCAGTACCGTTTTAGAGAG	Chr1: 74570094	Chr1:74570200
Dusp15	CGGGAGGCAGAAGAAAGTCTGCTTG	GGGTTCCGGGAGATGTTAGGACCTG	Chr2:152768947	Chr2:152769370
