# Supplementary material for: Multiplex Chromosomal Exome Sequencing Accelerates Identification of ENU-Induced Mutations in the Mouse
Source: G3 (Bethesda). 2012 Jan 1;2(1):143–50. doi: 10.1534/g3.111.001669 (PMC3276189; doi:10.1534/g3.111.001669)
Supplement: HTML Page - index.htslp [file supp_2.1.143_TableS2.pdf]

Table S2 Summary of embryos genotyped

Line	Litter	Wild type	Mutant	Abnormal
AB5	33	201	57	39
M2	41	254	80	9
X5	32	161	55	34
Y1	33	196	42	21
